# Modulation of Regulatory T Cells Activity by Distinct CD80 and CD86 Interactions With CD28/CTLA-4 in Chagas Cardiomyopathy

**DOI:** 10.3389/fcvm.2022.750876

**Published:** 2022-05-19

**Authors:** Bruna F. Pinto, Nayara I. Medeiros, Andrea Teixeira-Carvalho, Jacqueline A. Fiuza, Silvana M. Eloi-Santos, Maria C. P. Nunes, Silvana A. Silva, Tereza C. M. Fontes-Cal, Mayara Belchior-Bezerra, Walderez O. Dutra, Rodrigo Correa-Oliveira, Juliana A. S. Gomes

**Affiliations:** ^1^Departamento de Morfologia, Laboratório de Biologia das Interações Celulares, Instituto de Ciências Biológicas, Universidade Federal de Minas Gerais, Belo Horizonte, Brazil; ^2^Instituto René Rachou, Fundação Oswaldo Cruz–FIOCRUZ, Belo Horizonte, Brazil; ^3^Departamento de Clínica Médica, Faculdade de Medicina, Universidade Federal de Minas Gerais, Belo Horizonte, Brazil; ^4^Instituto Nacional de Ciência e Tecnologia Doenças Tropicais, Belo Horizonte, Brazil

**Keywords:** Chagas disease, CD80 co-stimulatory molecule, Treg cells, immune response, Chagas cardiomyopathy

## Abstract

Chagas cardiomyopathy is the symptomatic cardiac clinical form (CARD) of the chronic phase of Chagas disease caused by *Trypanosoma cruzi* infection. It was described as the most fibrosing cardiomyopathies, affecting approximately 30% of patients during the chronic phase. Other less frequent symptomatic clinical forms have also been described. However, most patients who progress to the chronic form develop the indeterminate clinical form (IND), may remain asymptomatic for life, or develop some cardiac damage. Some mechanisms involved in the etiology of the clinical forms of Chagas disease have been investigated. To characterize the contribution of CD80 and CD86 co-stimulatory molecules in the activation of different CD4^+^ (Th1, Th2, Th17, and Treg) and CD8^+^ T lymphocyte subsets, we used blocking antibodies for CD80 and CD86 receptors of peripheral blood mononuclear cells (PBMC) in cultures with *T. cruzi* antigens from non-infected (NI), IND, and CARD individuals. We demonstrated a higher frequency of CD8^+^ CD25^+^ T lymphocytes and CD8^+^ Treg cells after anti-CD80 antibody blockade only in the CARD group. In contrast, a lower frequency of CD4^+^ Treg lymphocytes after anti-CD86 antibody blockade was found only in IND patients. A higher frequency of CD4^+^ Treg CD28^+^ lymphocytes, as well as an association between CD4^+^ Treg lymphocytes and CD28^+^ expression on CD4^+^ Treg cells in the CARD group, but not in IND patients, and once again only after anti-CD80 antibody blockade, was observed. We proposed that Treg cells from IND patients could be activated *via* CD86-CTLA-4 interaction, leading to modulation of the immune response only in asymptomatic patients with Chagas disease, while CD80 may be involved in the proliferation control of T CD8^+^ lymphocytes, as also in the modulation of regulatory cell activation *via* CD28 receptor. For the first time, our data highlight the role of CD80 in modulation of Treg lymphocytes activation in patients with CARD, highlighting a key molecule in the development of Chagas cardiomyopathy.

## Introduction

Chagas disease, also known as American trypanosomiasis, is a neglected tropical parasitic disease caused by the protozoan *Trypanosoma cruzi* (1), affecting approximately 8 million people worldwide (2). The increase in morbidity in non-endemic regions, as well as the resurgence of transmission in endemic countries, is currently becoming a major focus of attention (3).

During the chronic phase, approximately 60% of patients do not develop specific clinical symptoms of the disease, classified as indeterminate clinical form (IND) (4–7). They can remain asymptomatic throughout their lives (8). However, in the 10–20 years of range, IND patients are likely to develop some cardiac damage (8, 9). Another 30% of infected individuals develop severe cardiomyopathy with progressive myocardium damage due to inflammation and fibrosis (4, 7). Heart failure caused by chronic Chagas disease has the worst prognosis among other cardiomyopathies (4, 7, 10, 11).

Some mechanisms involved in the etiology of the clinical forms of Chagas disease have been investigated, but even 112 years after the first description of this morbidity, science still does not understand why different clinical manifestations are developing in the chronic phase of the infection. The study of the immune response is crucial to determine the development of the disease by mobilizing multiple humoral and cellular mechanisms of the innate and acquired immune response (12, 13).

During an acute *T. cruzi* infection, antigen-presenting cells, such as monocytes, activate CD4^+^ T lymphocytes and initiate IL-12 secretion, which is the primary driver of IFN-γ-producing T helper 1 (Th1) cell differentiation. Th1 is essential for controlling the replication of the parasite but not for completely eliminating them, which contributes to the establishment of low-grade chronic persistent infection (7, 10, 14). While IND patients develop mechanisms to regulate the immune response, such as IL-10 production and Treg cell activation, CARD patients have a strong Th1-lymphocyte. This Th1 mechanism contributes to high levels of IFN-γ and TNF-α pro-inflammatory cytokines that lead to an exacerbated and uncontrolled inflammatory response (15–19).

*Trypanosoma cruzi* infection also leads to CD8 T^+^ lymphocytes activation, which shows cytotoxic activity by destroying infected cells with *T. cruzi* amastigotes, as well as through the production of IFN-γ for infection control (20–22). Studies with cardiac tissue biopsy fragments from patients with CARD have shown a high frequency of T cells in the inflammatory infiltrate, mainly CD8^+^ T lymphocytes, which contributes to cardiac fibers destruction, fibrosis development, and tissue damage (23–25).

Two signals are required for T CD4^+^ and T CD8^+^ lymphocyte activation. The first is mediated by the MHC and TCR receptors and the second by the CD80 and CD86 co-stimulating molecules that interact with the CD28 or CTLA-4 receptors present in the lymphocyte cell surface (26, 27). The interaction of the TCR receptor with the MHC-bound peptide is not sufficient for lymphocyte activation and a second activation signal is required (26, 28, 29). The interaction of CD80 or CD86 with CD28 leads to lymphocyte proliferation, and the interaction with CTLA-4 leads to inhibition of lymphocyte activity and thus downregulates the immune response (27, 30–32). CD28 receptor is constitutively expressed on T cells, has low avidity for CD80 and CD86 molecules and when activated, it triggers strongly up-regulates T cell cytokine production (26, 27, 33, 34). On the contrary, CTLA-4 has high avidity for CD80 and CD86 molecules, and its expression is rapidly increased only after T cell activation (33, 35). CTLA-4-deficient mice showed elevated leukocyte infiltrate in various organs, pointing to the role of CTLA-4 as a negative regulator of T cell activation (36). In Chagas disease, it was observed that there was a higher frequency of CD80^+^ monocytes in IND and CARD patients and a lower frequency of CD86^+^ monocytes only in the CARD form (30, 37, 38). The IND group also showed a higher CTLA-4 expression on T lymphocytes after exposure of monocytes to the Y strain of *T. cruzi* (30).

Although the expression of these co-stimulating molecules is essential for the activation or inhibition of lymphocytes (39–41), few studies to date have demonstrated the capacity of these receptors to activate CD4^+^ and CD8^+^ T lymphocytes in Chagas disease (38, 42). We previously demonstrated that CD86 may be involved in immunoregulation by CTLA-4 association, suggesting a strategy to control inflammation and tissue damage in IND patients (38).

This study characterizes the contribution of CD80 and CD86 co-stimulatory molecules in the activation of CD4^+^ subpopulations (Th1, Th2, Th17, and Treg) and CD8^+^ T lymphocyte subsets by blocking antibodies to inhibit CD80 and CD86 receptors of peripheral blood mononuclear cells (PBMC) in cultures with *T. cruzi* antigens. We demonstrated that CD80 and CD86 receptors have different functions in lymphocytes activation from patients with Chagas disease, and CD80 may be responsible for the modulation of Treg lymphocytes activation in patients with CARD, pointing out a key molecule in the development of cardiomyopathy.

## Materials and Methods

### Study Population

The patients who agreed to participate in this study were selected at the outpatient clinic of the Ambulatório de Doenças infecto-parasitárias Alda Falcão in Instituto René Rachou and Ambulatório Bias Fortes of the Hospital das Clinicas of Universidade Federal de Minas Gerais, Belo Horizonte, Minas Gerais, Brazil.

Serology for Chagas disease was defined by the following tests: indirect immunofluorescence, ELISA, or indirect hemagglutination, and patients were considered infected when they tested positive for at least two different serological tests. In total, 20 patients with Chagas disease were grouped as indeterminate (IND) and cardiac (CARD) clinical forms. The IND group (*n* = 9) included asymptomatic individuals who tested positive for Chagas disease but did not have significant alterations in electrocardiography, echocardiogram, esophagogram, chest X-ray, and barium enema. The CARD group (*n* = 11) was represented by patients with alterations in electrocardiography who were already monitored by the responsible physicians at Ambulatório de Doenças infecto-parasitárias Alda Falcão in Instituto René Rachou and Ambulatório Bias Fortes of the Hospital das Clinicas of Universidade Federal de Minas Gerais. Left ventricular end-diastolic diameter/body surface area ≥31 mm (64.8 ± 5.9 mm) and left ventricular ejection fraction <55% (34 ± 10%) were used as echocardiographic parameters of Chagas dilated cardiomyopathy. The non-infected group (NI, *n* = 9) included normal, healthy individuals from non-endemic areas for Chagas disease who tested negative for *T. cruzi* infection. The age of the patients included in this study was between 30 and 75 years.

### *Trypanosoma cruzi* Soluble Antigen Preparations

Tissue culture-derived trypomastigotes of the CL-Brener strain of *T. cruzi* were isolated from LLC cells maintained in RPMI 1640 medium (Gibco, Thermo Fisher Scientific, United States) supplemented with 10% fetal bovine serum, as previously described (43). Later, trypomastigotes were collected from the supernatant and were lysed and homogenized by a glass homogenizer and Teflon pestle in cold phosphate-buffered saline (PBS, Sigma, United States). Next, the suspensions were centrifuged at 23,000 × *g* for 60 min at 4°C, and the supernatant was collected, dialyzed for 24 h at 4°C against PBS, and sterilized by filtration on 0.2 μm pore size membranes. The protein concentration was measured by Nanodrop (Thermo Scientific, United States), and the material was separated into aliquots and stored at −70°C. Parasites obtained were used to infect PBMC from patients and healthy individuals at a final concentration of 20 μg/ml.

### Obtaining Peripheral Blood Mononuclear Cells

The peripheral blood of the individuals was collected in a sterile Vacutainer tube containing heparin and was slowly added over Ficoll-Hypaque (Sigma) in a proportion of 1:1 in a 14-ml polypropylene tube (Falcon, United States). It was centrifuged at 400 × *g* for 40 min at room temperature, and, at the end of the centrifugation, peripheral blood mononuclear cells (PBMC) ring was obtained between the Ficoll-Hypaque mixture and the plasma. The plasma was carefully removed, subsequently stored in 2 ml aliquots, and duly identified at −20°C. The PBMC was removed with the aid of a micropipette and transferred to a 50-ml Falcon tube. The cells were washed three times by centrifugation at 400 × *g* for 10 min at 4*^o^*C in sterile PBS and resuspended in RPMI 1640 medium to 10^7^ cells/ml. All manipulations were performed under sterile conditions in a laminar flow hood.

### Anti-CD80 and Anti-CD86 Blocking Assay

To perform co-stimulation blockade by anti-CD80 and anti-CD86 antibodies, CMBLAST medium was used, containing 1.6% L-glutamine (Sigma), 3% penicillin and streptomycin, and 5% inactivated AB-type human serum (Sigma) diluted in RPMI 1640 medium. PBMC culture was carried out in polypropylene tubes of 5 ml at a concentration of 10^7^ cells/ml with CMBLAST medium. TRYPO (20 μg/ml), anti-CD80 monoclonal antibody blockade (2D10.4, 5 μg/ml), or anti-CD86 monoclonal antibody blockade (IT2.2, 5 μg/ml) (Thermo Fisher Scientific, Waltham, MA, United States) were added to the tubes, obtaining a final volume of 1.5 ml. As a control, the same conditions described above were used in tubes in the absence of TRYPO stimulation, as well as in the absence of anti-CD80 or anti-CD86 monoclonal antibody blockade. The tubes were incubated for 18 h at 37°C under 5% CO_2_ (Forma Scientific, United States).

### Flow Cytometry

After *in vitro* assay, Brefeldin-A (1 mg/ml [Sigma, United States]) was added to the cells, incubated for 4 h, and then washed in sterile PBS. Brefeldin-A is a blocker of protein trafficking to the Golgi complex, leading to the accumulation of intracellular proteins and being a potent inhibitor of cell secretion (44). The cells were transferred to 5-ml polystyrene tubes containing the corresponding surface antibodies and were incubated for 30 min in the dark at room temperature. Next, cells were washed with PBS-W (PBS pH 7.4, containing 0.5% BSA and 0.1% sodium azide) and were fixed with 2% formaldehyde, followed by incubation for 20 min in the dark at room temperature. A total of 2.5 ml of PBS-P (PBS, pH 7.4 containing 0.5% BSA, 0.1% sodium azide, and 0.5% saponin) were added, and tubes were incubated for 15 min in the dark at room temperature. To evaluate intracellular markers, 2 μl of anti-cytokine antibody was added to each tube and incubated for 1 h at room temperature. Then, cells were washed with 1 ml of PBS-P, 150 μl of PBS was added to the tubes, and the samples containing the cell suspension were used to acquire data on a flow cytometer (FACS LSR Fortessa, BD, United States). A total of 50,000 events were analyzed using size (FSC) and granularity (SSC) parameters. Monoclonal antibodies were conjugated to FITC, PERCP (or PE-Cy5 or PERCP-Cy5.5), APC, APCCy7, PE-Cy7, and BV421. The analyzed molecules (clones) were CD4 (RPA-T4), CD28 (CD28.2), CTLA-4 (BNI3), CD25 (M-A251), HLA-DR (G46-6), CD80 (2D10), CD86 (2331), Tbet (4B10), GATA-3 (L50-823), RORγT (Q21-559), FOXP3 (PCH101), IFN-γ (25723.11), IL-4 (8D4-8), IL-10 (JES3-19F1), and IL-17 (eBio64DEC17).

### Statistical Analysis

For each co-stimulatory molecule blockade antibody (anti-CD80 and anti-CD86), four treatments were prepared: (A) only medium (control); (B) medium with TRYPO; (C) medium with blockade antibody; and (D) medium with TRYPO and blockade antibody.

The data in each NI, IND, and CARD group were normalized using ratio calculation of the four treatments. First, the values obtained in (C) treatment were divided by (A) treatment values. This ratio (C/A) demonstrates the effect of co-stimulatory molecules blockade in cells. Second, the values obtained in the (D) treatment were divided by (B) treatment values. This ratio (D/B) demonstrates the effect of co-stimulatory molecules blockade in cells and the effects of stimulation with TRYPO. Finally, from a new division [(D/B)/(C/A)], we obtained a normalized quotient, corresponding to the effect of co-stimulatory molecules blockade and the TRYPO stimulation, nullifying the medium variations.

To analyze the pattern of cytokines expressed by CD4^+^ T lymphocytes (IFN-γ, IL-4, IL-17, and IL-10), we calculated a relative expression index through the ratio between the effect of anti-CD86 blockade and the effect of anti-CD80 blockade in TRYPO stimulation. For the analysis of cytokines and the proportion of co-stimulatory molecules expressed by lymphocyte subsets, raw data obtained from treatments only after TRYPO stimulation (treatments B and D) were used.

Afterward, the statistical analysis was performed comparing the quotient of each NI, IND, and CARD group. All data assumed a non-Gaussian distribution. Differences between groups were analyzed by non-parametric Kruskal–Wallis test followed by Dunn’s multiple comparisons test, using the GraphPad Prism version 5.0 software (San Diego, United States). Paired analysis through Wilcoxon signed-rank test was used to verify differences between the total groups (NI, IND, and CARD, *n* = 29) before and after anti-CD86 blockade on anti-CD80 antibody blockade.

The association between Treg lymphocytes and CD28 and CTLA-4 receptors was determined from the linear regression, considering the coefficient of determination (R^2^) for the quality of fit and the *F* test to measure the variance between pairs (*p* < 0.05). For all analyses, the confidence interval was 95%, and significant statistical differences were considered when *p* < 0.05.

## Results

### The Use of Blocking Monoclonal Antibodies Reduces the Frequency and Mean Fluorescence Intensity of CD80 and CD86 Receptors, Respectively

Although the role of CD80 and CD86 co-stimulatory molecules is already known to activate adaptive immunity, the involvement of these molecules in Chagas disease is poorly studied. Thus, using monoclonal blocking antibody for CD80 and CD86 receptors, we assessed the functional phenotypic profile of CD4^+^ T and CD8^+^ T lymphocytes and their subsets from cultures of PBMC after TRYPO stimulation from healthy and Chagas disease patients.

**FIGURE 1:**
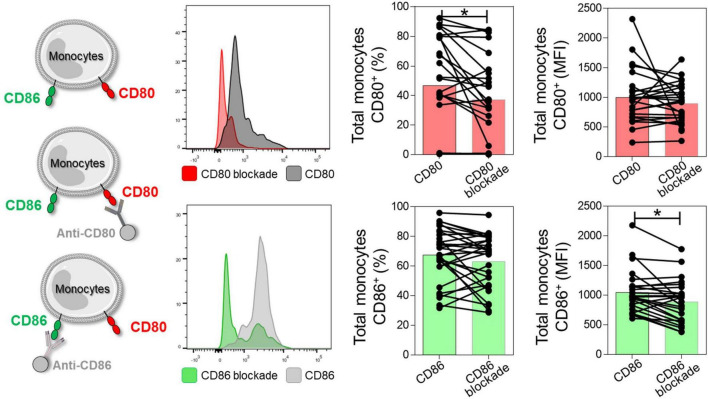
Representation of the reduction of CD80 and CD86 molecules after antibody blockade. Histogram representation, frequency of CD80^+^ or CD86^+^ monocytes, and mean fluorescence intensity (MFI) after and before anti-CD80 and anti-CD86 antibody blockade of PBMC cultures from total group (NI, IND, and CARD, *n* = 29). For this analysis, monocytes were delimited through granularity (SSC) vs. HLA-DR parameters and subsequent evaluation of the CD80 and CD86 co-stimulatory molecules. Statistical differences (*p* < 0.05) were obtained by Wilcoxon signed-rank test. Bars represent the median values.

First, to validate the anti-CD80 and anti-CD86 blocking assay, we evaluated the expression of CD80 and CD86 co-stimulatory molecules before and after anti-CD80 and anti-CD86 antibody blockade in PBMC culture from NI, IND, and CARD groups. Reduction in the frequency of total monocytes CD80^+^ and mean fluorescence intensity (MFI) of CD86 were observed after anti-CD80 and anti-CD86 antibody blockade, respectively (Figure 1).

### CD86 Contributes to Control the Inflammatory Response While CD80 Has a Protective Profile Only in Asymptomatic Patients With Chagas Disease

**FIGURE 2:**
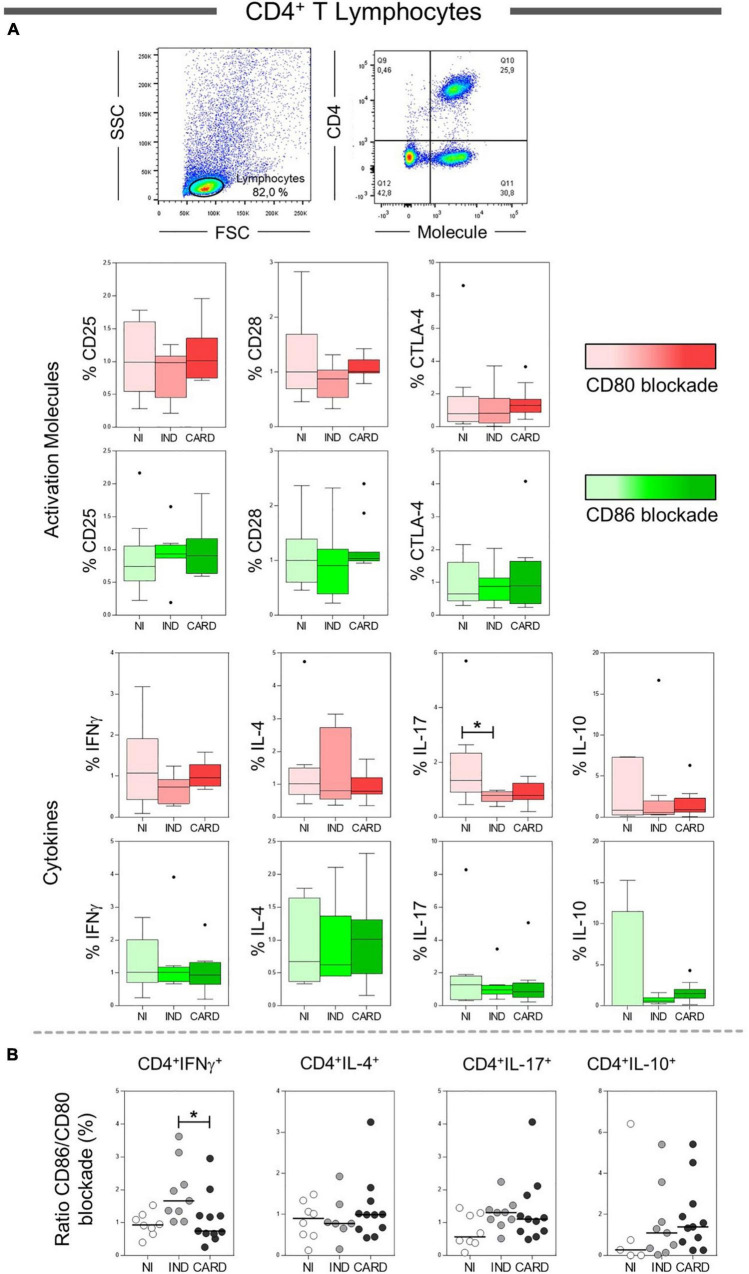
Analysis of the phenotypic-functional profile of CD4^+^ T lymphocytes after anti-CD80 and anti-CD86 antibody blockade. Flow cytometry gate strategy of non-infected (NI) individuals is represented. Frequency of T CD4^+^ lymphocytes expressing CD25, CD28, and CTLA-4 activations molecules and IFN-γ, IL-4, IL-17, and IL-10 cytokines **(A)** in PBMC culture from NI individuals (*n* = 9), indeterminate (IND, *n* = 9), and cardiac (CARD, *n* = 11) clinical forms of Chagas disease, after TRYPO *in vitro* stimulation and anti-CD80 and anti-CD86 antibody blockade. Relative expression index of IFN-γ, IL-4, IL-17, and IL-10 cytokines through CD86/CD80 blockade ratio by CD4^+^ T lymphocytes **(B)**. Statistical differences (*p* < 0.05) between groups were obtained by Kruskal–Wallis test, followed by Dunn’s post-test, and represented by asterisk (*) and lines. Boxes demonstrate the median and interquartile ranges, whiskers evidence the highest and lowest observation, and dots represent the outliers.

To assess the effect of CD80 and CD86 co-stimulatory molecules on the activation or inhibition of total CD4^+^ T lymphocytes, we analyzed the relationship between these molecules and their CD28 and CTLA-4 ligands. However, no difference was observed in these molecules. In addition, we evaluated the main cytokines produced by total CD4^+^ T cells after anti-CD80 or anti-CD86 antibody blockade from PBMC cultures of Chagas disease patients and healthy individuals. We observed a significant reduction in the frequency of CD4^+^ IL-17^+^ T lymphocytes in IND patients when compared to the NI group only in the presence of anti-CD80 antibody blockade (Figure 2A). Other significant differences were not observed.

Next, to analyze the pattern of cytokines expressed by CD4^+^ T lymphocytes (IFN-γ, IL-4, IL-17, and IL-10), we calculated a relative expression index through the ratio between anti-CD86/anti-CD80 blockade from PBMC cultures of NI, IND, and CARD groups. Our data demonstrated a higher frequency of CD4^+^ IFN-γ^+^ T lymphocytes after anti-CD86 antibody blockade compared to anti-CD80 antibody blockade in IND compared to patients with CARD (Figure 2B). Other significant differences were not observed.

### CD8^+^ T Lymphocytes Activation Could Be Modulated by CD80 Co-stimulatory Molecule

**FIGURE 3:**
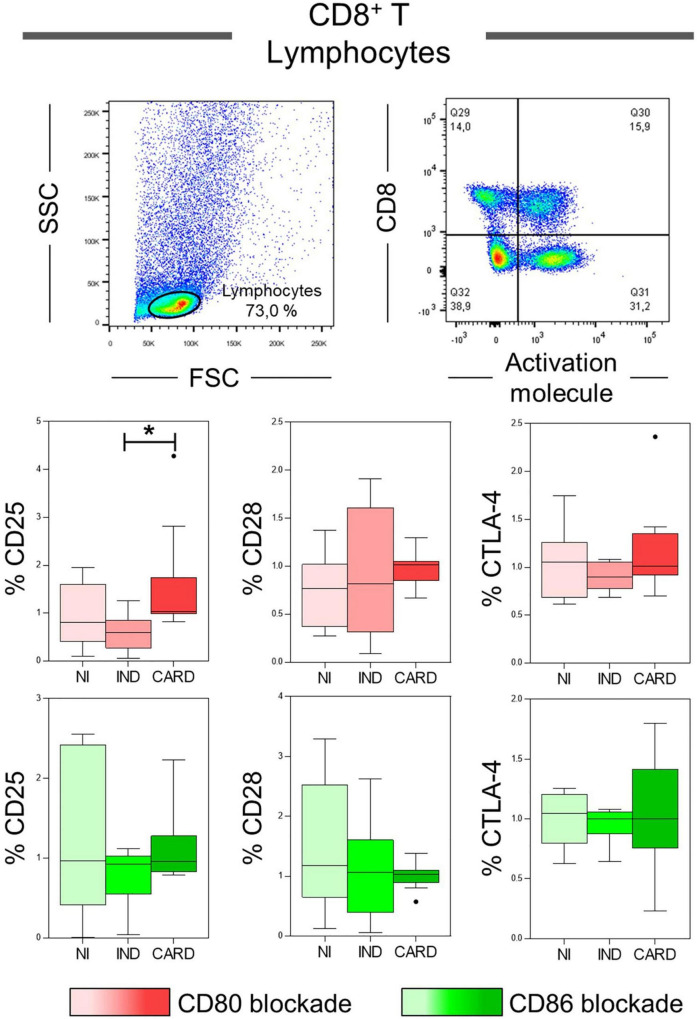
Analysis of the phenotypic-functional profile of CD8^+^ T lymphocytes after anti-CD80 and anti-CD86 antibody blockade. Flow cytometry gate strategy of non-infected (NI) individuals is represented. Frequency of T CD8^+^ lymphocytes expressing CD25, CD28, and CTLA-4 activations molecules in PBMC culture from NI individuals (*n* = 9), indeterminate (IND, *n* = 9), and cardiac (CARD, *n* = 11) clinical forms of Chagas disease, after *in vitro* stimulation with *T. cruzi* antigens and anti-CD80 and anti-CD86 antibody blockade. Statistical differences (*p* < 0.05) between groups were obtained by Kruskal-Wallis test, followed by Dunn’s post-test, and represented by asterisk (*) and lines. Boxes demonstrate the median and interquartile ranges, whiskers evidence the highest and lowest observation, and dots represent the outliers.

CD8^+^ T lymphocytes play a crucial role during the acute and chronic phases of Chagas disease, and, considering that their activation may be dependent of CD80 and CD86 molecules performance (20, 45, 46), our next step was to evaluate the expression of the CD25, CD28, and CTLA-4 activation molecules in total CD8^+^ T lymphocytes after anti-CD80 or anti-CD86 antibody blockade of NI, IND, and CARD groups. The results showed a higher frequency of CD8^+^ CD25^+^ T lymphocytes in the CARD group when compared to the IND group only after anti-CD80 antibody blockade (Figure 3). Other significant differences were not observed.

### CD80 and CD86 Modulate Regulatory T Cells Activity in Cardiac and Indeterminate Patients, Respectively

**FIGURE 4:**
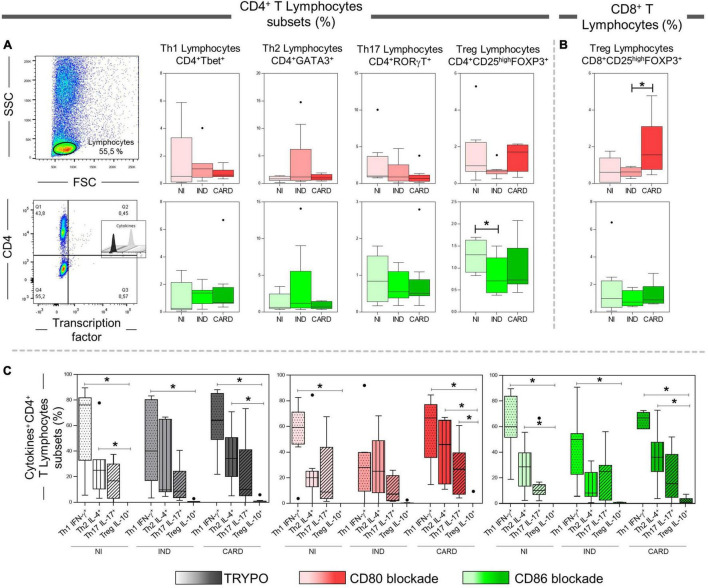
Analysis of CD4^+^ and CD8^+^ T lymphocytes subsets after anti-CD80 and anti-CD86 antibody blockade and pattern of expressed cytokines in PBMC cultures with TRYPO, CD80, or CD86 antibody blockade. Flow cytometry gate strategy of non-infected (NI) individuals is represented. Frequency of Th1, Th2, Th17, and CD4^+^ Treg lymphocytes **(A)** and CD8^+^ Treg lymphocytes **(B)** after *in vitro* stimulation with *T. cruzi* antigens and anti-CD80 and anti-CD86 antibody blockade. Th1 IFN-γ^+^, Th2 IL-4^+^, Th17 IL-17^+^, and CD4^+^ Treg IL-10^+^ PBMC cultures with TRYPO, with anti-CD80 antibody blockade, and with anti-CD86 antibody blockade **(C)** from NI individuals (*n* = 9), indeterminate (IND, *n* = 9), and cardiac (CARD, *n* = 11) clinical forms of Chagas disease. Statistical differences (*p* < 0.05) between groups were obtained by Kruskal–Wallis test, followed by Dunn’s post-test, and represented by asterisk (*) and lines. Boxes demonstrate the median and interquartile ranges, whiskers evidence the highest and lowest observation, and dots represent the outliers.

After anti-CD80 and anti-CD86 blocking assays on CD4^+^ and CD8^+^ T total lymphocytes, we wondered what role these co-stimulatory molecules play in activating the CD4^+^ and CD8^+^ T subsets. The data show a lower frequency of CD4^+^ Treg cells in IND when compared to the NI group only with anti-CD86 antibody blockade (Figure 4A). In contrast, the CARD group showed a higher frequency of CD8^+^ Treg lymphocytes in comparison with IND individuals only in PBMC cultures after anti-CD80 antibody blockade (Figure 4B). Other significant differences were not observed.

Subsequently, we evaluated the expression of IFN-γ, IL-4, IL-17, and IL-10 cytokines by Th1, Th2, Th17, and CD4^+^ Treg lymphocytes, respectively, in PBMC cultures with TRYPO, with anti-CD80 antibody blockade, and with anti-CD86 antibody blockade from NI, IND, and CARD groups. A lower frequency of Treg IL-10^+^ lymphocytes when compared to Th1 IFN-γ^+^ cells in the three PBMC cultures and from all groups evaluated was observed, except in the case of the IND group after CD80 blockade. Our results also showed a reduction in the frequency of Treg IL-10^+^ cells in comparison with Th2 IL-4^+^ lymphocytes in PBMC cultures with TRYPO and with anti-CD86 antibody blockade from NI and CARD groups, as well as in PBMC culture with anti-CD80 antibody blockade from the CARD group. In addition, only in PBMC culture with anti-CD80 antibody blockade from the CARD group was observed lower frequency of Treg IL-10^+^ lymphocytes when compared to Th17 IL-17^+^ cells (Figure 4C). Other significant differences were not observed.

### CD80 Appears to Modulate Treg Activation Only in Cardiac Patients *via* CD28 Receptor

**FIGURE 5:**
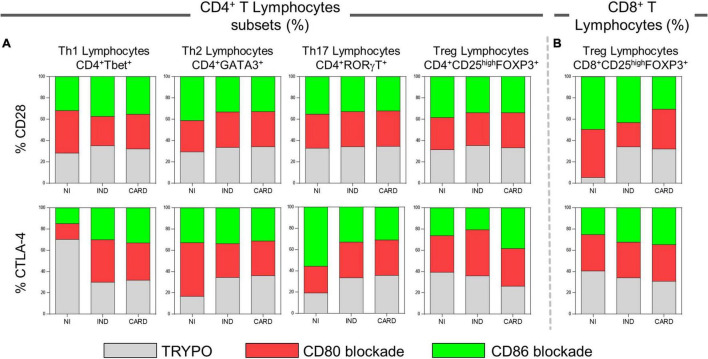
Proportion of CD28 and CTLA-4 expression by lymphocyte subsets. Proportion of CD28 and CTLA-4 ligands on Th1, Th2, Th17, CD4^+^ Treg **(A)**, and CD8^+^ Treg lymphocytes **(B)** in PBMC cultures with TRYPO, with anti-CD80 antibody blockade, and with anti-CD86 antibody blockade from NI individuals (*n* = 9), indeterminate (IND, *n* = 9), and cardiac (CARD, *n* = 11) clinical forms of Chagas disease.

We evaluated the proportion of CD28 and CTLA-4 ligands in each lymphocyte subsets in PBMC cultures with TRYPO, with anti-CD80 antibody blockade, and with anti-CD86 antibody blockade from NI, IND, and CARD individuals. In patients with Chagas disease, regardless of the clinical form, the same proportion of CD28 on Th1, Th2, Th17, and CD4^+^ Treg was observed in the three cultures of PBMC evaluated. In contrast, we found a higher proportion of CD8^+^ Treg CD28^+^ cells in PBMC culture with anti-CD80 antibody blockade from CARD patients compared to PBMC culture with anti-CD86 antibody blockade and to IND group. When we analyzed the frequency of CTLA-4, we observed a similar proportion of this ligand on the Th1, Th2, Th17, and CD8^+^ Treg lymphocyte subsets of Chagas disease patients. On the contrary, lower proportion of CD4^+^ Treg CTLA-4^+^ lymphocytes was observed only in PBMC culture with anti-CD86 antibody blockade from IND group in comparison with PBMC culture with anti-CD80 antibody blockade and the CARD group (Figure 5).

**FIGURE 6:**
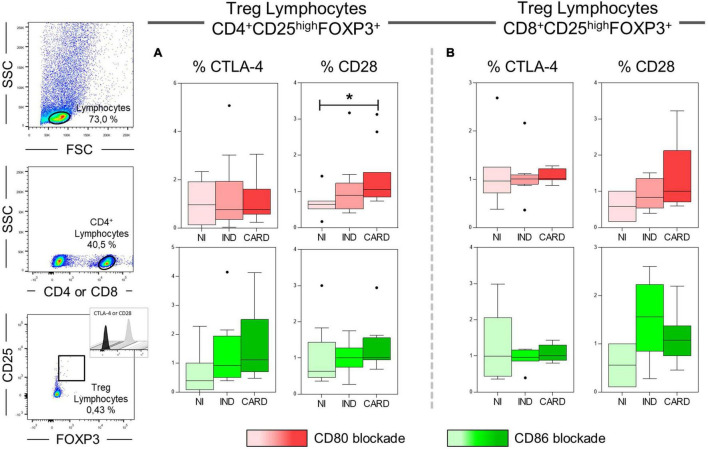
Analysis of the frequency of CD28 and CTLA-4 ligands on Treg lymphocytes after anti-CD80 and anti-CD86 antibody blockade. Flow cytometry gate strategy of non-infected (NI) individuals is represented. Frequency of T CD4^+^ Treg lymphocytes **(A)** and CD8^+^ Treg lymphocytes **(B)** expressing CD28 and CTLA-4 in PBMC culture from NI individuals (*n* = 9), indeterminate (IND, *n* = 9), and cardiac (CARD, *n* = 11) clinical forms of Chagas disease, after *in vitro* stimulation with *T. cruzi* antigens and anti-CD80 and anti-CD86 antibody blockade. Statistical differences (*p* < 0.05) between groups were obtained by Kruskal–Wallis test, followed by Dunn’s post-test, and represented by asterisk (*) and lines. Boxes demonstrate the median and interquartile ranges, whiskers evidence the highest and lowest observation, and dots represent the outliers.

Our next step was to assess significantly the expression of CTLA-4 and CD28 ligands on CD4^+^ and CD8^+^ Treg lymphocytes after anti-CD80 or anti-CD86 antibody blockade in PBMC cultures of NI, IND, and CARD individuals. Our results showed a higher frequency of CD4^+^ Treg CD28^+^ lymphocytes in CARD in comparison with NI group only after anti-CD80 antibody blockade (Figure 6). Other significant differences were not observed.

**FIGURE 7:**
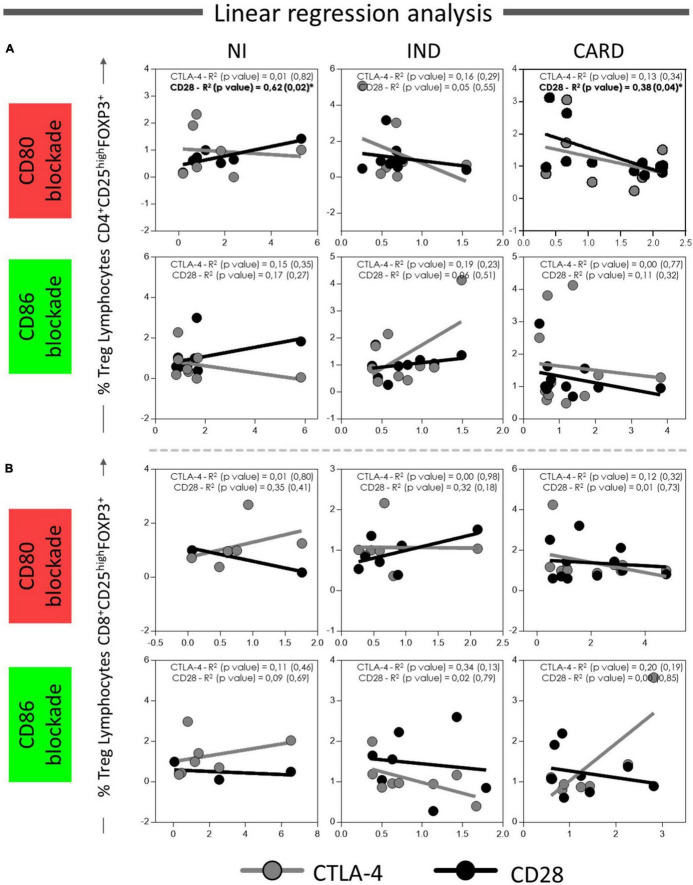
Linear regression analysis between CD4^+^ and CD8^+^ Treg lymphocytes and CD28 and CTLA4 ligands on Treg lymphocytes. Association between CD4^+^ Treg lymphocytes **(A)** and CD8^+^ Treg lymphocytes **(B)** with CD28 and CTLA-4 expressed in CD4^+^ and CD8^+^ Treg cells from PBMC culture of NI individuals (*n* = 9), indeterminate (IND, *n* = 9), and cardiac (CARD, *n* = 11) clinical forms of Chagas disease, after *in vitro* stimulation with *T. cruzi* antigens and anti-CD80 and anti-CD86 antibody blockade. Data are presented by R-squared (R^2^), and significant statistical differences were considered when *p* < 0.05.

Finally, to understand the possible relationship between CD80 and CD86 co-stimulatory molecules and CD28 and CTLA-4 expressed in CD4^+^ and CD8^+^ Treg lymphocytes, we investigated the interaction between these molecules after anti-CD80 or anti-CD86 antibody blockade of NI, IND, and CARD groups through linear regression analysis. We found a significant association between CD4^+^ Treg lymphocytes and CD4^+^ Treg CD28^+^ cells in NI (*R*^2^ = 0.62/*p* = 0.02) and CARD (*R*^2^ = 0.38/*p* = 0.04) groups, but not in IND patients, and only in PBMC cultures after anti-CD80 blockade (Figure 7). Other significant differences were not observed.

## Discussion

Inflammatory stimuli modulate the expression of CD80 and CD86 co-stimulatory ligands, resulting in the activation or attenuation of signals that determine the nature and development of the subsequent immune response (32). It has been demonstrated that the absence of the second signal mediated by CD80 and CD86 to T cell activation drives lymphocyte anergy (26), and the simultaneous blockade of these both molecules results in exacerbated *T. cruzi* infection in a murine model (47). Therefore, CD80 and CD86 co-stimulatory molecules play a critical role in the control of the immune responses. However, little is known about the role of these molecules in activating adaptive immunity mediated by CD4^+^ and CD8^+^ T lymphocytes and their subsets during the chronic phase of Chagas disease. We previously described that IND and CARD patients differentially expressed CD80 and CD86 co-stimulatory molecules, and we proposed that CD86 may be involved in the immunomodulation in asymptomatic patients (38). Here, we confirmed the regulatory performance of CD86 and highlighted the role of the CD80 molecule in the regulation of Treg activation in patients with CARD.

We evaluated the role of CD80 and CD86 molecules in targeting CD4^+^ T lymphocytes, and we found that blocking the CD80 receptor led to a reduction in the frequency of CD4^+^IL-17^+^ T lymphocytes in IND patients. It has been proposed that IL-17 is associated with better cardiac function in patients with Chagas disease (48, 49) and this cytokine may regulate the immune response and the development of cardiac lesions during *T. cruzi* infection (50). On the contrary, our results showed that only after anti-CD86 antibody blockade, higher frequency of CD4^+^ IFN-γ^+^ T lymphocyte was observed also in the IND group. IFN-γ production is required to control replication of the parasite during the acute phase of *T. cruzi* infection, even as contributing to the increase of the inflammatory process during the chronic phase (12, 15, 51). Thereby, we proposed that CD86 can control IFN-γ^+^ expression by CD4^+^ T cells in asymptomatic patients, regulating the development of exacerbated inflammation, and CD80 displays a protective role through the regulation of IL-17, participating in myocardium tissue homeostasis only in IND patients.

We demonstrated a higher frequency of CD8^+^ CD25^+^ T lymphocytes and CD8^+^ Treg cells in the CARD group after anti-CD80 antibody blockade in contrast with lower frequency of CD4^+^ Treg lymphocytes after anti-CD86 antibody blockade found only in IND patients. It has been proposed that even IND patients has the highest frequency of Treg cells in peripheral blood and these cells was associated with a better clinical prognosis in patients with the asymptomatic clinical form of Chagas disease (19, 38, 52), CARD patients also have higher frequency of Treg lymphocytes in comparison with NI individuals (38). Thus, even though patients with CARD present an imbalance in the production of effector and immunoregulatory mechanisms that contribute to the worsening of myocardial damage, these individuals are able to produce regulatory T lymphocytes to try to control inflammation. On the contrary, the production of Treg lymphocytes in IND patients indeed appears to be a key factor in regulating the inflammatory process, preventing tissue damage. Here, we proposed that these regulatory cells can be activated in both IND and CARD clinical forms of chronic Chagas disease by opposite co-stimulation molecules. We have previously demonstrated an association between CD86 receptor expressed in non-classical monocytes with Treg lymphocytes, while a negative correlation with CD80 by total monocytes and these regulatory cells was found in patients without cardiomyopathy (NI and IND), but not in CARD group (38). Moreover, as observed in this study, only anti-CD86 antibody blockade, but not CD80, led to a reduction of CD4^+^ Treg IL-10^+^ cells in IND patients. These findings suggest that while CD86, but not CD80, may contribute to the activation of Treg lymphocytes as well as the production of IL-10 by these cells only in IND individuals, leading to modulation of the immune response, in the CARD group, CD80 may be responsible for controlling CD8^+^ CD25^+^ T lymphocyte activation and could be involved in the modulation of Treg cell induction.

Activation or inhibition of T lymphocytes requires the interaction between CD80 and CD86 co-stimulatory molecules with their ligands CD28 and CTLA-4 that can direct the plasticity in T lymphocyte subset activation (32). Through comparing the proportion of CD28 and CTLA-4 ligands in each lymphocyte subsets between patients with Chagas disease, we observed a higher proportion of CD28 on CD8^+^ Treg cells only after anti-CD80 blockade from CARD in comparison with anti-CD86 blockade and to IND group. Moreover, lower proportion of CTLA-4 on CD4^+^ Treg lymphocytes was observed only after anti-CD86 blockade from IND in comparison with anti-CD80 antibody blockade and to CARD group. Interestingly, when we evaluated the statistical results, we found a higher frequency of CD4^+^ Treg CD28^+^ lymphocytes in CARD group only after anti-CD80 antibody blockade. Furthermore, we verified an association between CD4^+^ Treg lymphocytes and CD28^+^ expression on CD4^+^ Treg cells in CARD group, but not in IND patients, and once again only in after anti-CD80 antibody blockade. Previously, it has been demonstrated by our research group that there is an association between CD80 and CD28, and between CD86 and CTLA-4 receptors on total CD4^+^ T lymphocytes, as also, increased frequency of CD4^+^CTLA-4^+^ T lymphocytes in IND group (38). CTLA-4 is constitutively expressed in murine and human Tregs being a key molecule involved in Treg-mediated suppression (53–55). CTLA-4 interacts with CD80 and CD86 on professional antigen-presenting cell (APCs), such as monocytes, and captures these ligands in a process called trans-endocytosis (56). Thus, CD80 and CD86 become unavailable to interact with CD28, leading to indirect inhibition of total T lymphocytes. Furthermore, it has been demonstrated that CD25^+^CD4^+^ Treg cells from deficient CD28 mice exhibited suppressive activity, indicating that this molecule is dispensable for the Treg-mediated suppression (54). Thus, we suggested that this CTLA-4-mediated suppression mechanism could be used by Treg cells only in IND, but not by CARD patients, since Chagas cardiomyopathy patients demonstrated a higher frequency of Treg CD28^+^ lymphocytes and not CTLA-4, as shown by IND patients in our previous studies (38).

Therefore, we proposed that Treg cells from IND patients could be activated *via* CD86-CTLA-4 interaction, leading to modulation of the immune response only in asymptomatic patients with Chagas disease, while CD80 may be an important molecule capable of modulating the expression of CD28 in Treg lymphocytes only from patients with CARD. Nolan et al., evaluated the role of CD80 and CD86 in mice and human with sepsis and observed that upregulation of CD80 on circulating monocytes was associated with severity of illness, while CD86 appears to have a protective performance, suggesting a relatively anti-inflammatory role of this co-stimulatory molecule *in vivo* (57). In addition, positive regulation of CD80 directs the polarization of Th1 lymphocytes (58). Therefore, we suggested that CD80 may be involved in the proliferation control of T CD8^+^ lymphocytes, as well as in the modulation of regulatory cells activation *via* CD28 receptor.

Here, we highlighted for the first time the role of CD80 in modulation of Treg lymphocyte activation in CARD patients, maybe by performance with CD28 receptor, pointing out a key molecule in the development of Chagas cardiomyopathy. It is important to mention that even though the CD80 and CD86 blockade used here reduced statistically both the frequency and the expression of these receptors in human monocytes, the *in vitro* experiments performed are limited by not completely blocking these receptors. Thus, the use of other methodological approaches, such as molecular tools to repress the expression of these receptors or to block their actions can provide more information about the role of CD80 and CD86 in chagasic pathology. Therefore, further studies are still needed to understand the immunological mechanisms involved in establishing the different clinical forms of Chagas disease.

## Data Availability Statement

The raw data supporting the conclusions of this article will be made available by the authors, without undue reservation.

## Ethics Statement

The studies (0021.0438.438-10) involving human participants were reviewed and approved by the Ethics Committee of the René Rachou Institute, FIOCRUZ, Minas Gerais (CEPSH-IRR #15/2011). The patients/participants provided their written informed consent to participate in this study.

## Author Contributions

BP did the statistical analysis, figures, and wrote the study. NM and JG delineated the experiments and discussed the results. AT-C, WD, and RC-O contributed to the design of the study and discussions of the results. SE-S, MN, and SS select and lead clinical management of patients. BP, NM, JF, TF-C, and MB-B performed the experiments. JG contributed to the conception and coordinated the study. All authors contributed to manuscript revision, read, and approved the submitted version.

## Conflict of Interest

The authors declare that the research was conducted in the absence of any commercial or financial relationships that could be construed as a potential conflict of interest.

## Publisher’s Note

All claims expressed in this article are solely those of the authors and do not necessarily represent those of their affiliated organizations, or those of the publisher, the editors and the reviewers. Any product that may be evaluated in this article, or claim that may be made by its manufacturer, is not guaranteed or endorsed by the publisher.
